# Perinatal consumption of a thermally processed diet alters feeding behavior and has sex-dependent effects upon metabolism in later life in mice

**DOI:** 10.1016/j.fochms.2026.100375

**Published:** 2026-02-19

**Authors:** M.T. Nogueira Silva Lima, C. Delayre-Orthez, M. Howsam, P. Jacolot, C. Niquet-Léridon, A. Okwieka, P.M. Anton, M. Perot, A. Ghinet, C. Jouquand, C. Fradin, E. Boulanger, S. Jaisson, P. Gillery, F.J. Tessier

**Affiliations:** a U1167-RID-AGE-Facteurs de Risque et Déterminants Moléculaires des Maladies Liées au Vieillissement, Institut Pasteur de Lille, University Lille, Inserm, CHU Lille, F-59000 Lille, France; b Univ. Lille, lnserm, CHU Lille, JUNIA, U1352 - BioPrev - Bien vieillir: de l'inflammaging à la prévention, 59000 Lille, France; c Institut Polytechnique UniLaSalle, Université d'Artois, ULR 7519, Equipe PETALES, 60000 Beauvais, France; d University of Reims Champagne-Ardenne, Laboratory of Biochemistry and Molecular Biology, CNRS/URCA UMR 7369 MEDyC, Faculté de Médecine, 51095 Reims, France; e University Hospital of Reims, Laboratory of Biochemistry-Pharmacology-Toxicology, 51092 Reims, France; f Junia, Health and Environment, Laboratory of Sustainable Chemistry and Health, 59000 Lille, France

**Keywords:** Maillard reaction, Thermal processing, Micronutrient loss, Metabolic programming, Food preference

## Abstract

Intense thermal processing of food, industrial or domestic, alters the food matrix leading to degradation of thermolabile vitamins such as thiamine, and to the formation of color and aroma compounds via the Maillard reaction which influence palatability and consumer preference. In the literature, baked diets are used to mimic industrial food processing and to study protein glycation in animal models; however, the relationships between food matrix transformation, metabolic outcomes, and food preference remain poorly understood. We hypothesized that perinatal exposure to a thermally processed food may disrupt metabolism and influence food intake. We quantified nutritional losses and Maillard reaction products (MRP) and assessed food preference and energy metabolism in 8-week-old, male and female C57BL/6 mice exposed perinatally to a standard (STD) or a baked (BKD) diet under controlled conditions. Compared with a STD diet, BKD showed lower lysine (1.2-fold) and thiamine (∼7-fold) content, alongside higher levels of carboxymethyllysine (4.5-fold), hydroxymethylfurfural (3–5-fold), and 2-furanmethanol (up to 9-fold). Although intake did not differ during compulsory feeding, mice preferred STD, as indicated by a decrease in BKD intake. Male mice exhibited reductions in lean mass and increased plasma urea, consistent with altered protein metabolism. Females displayed increased leptin, TNFα, and leptin receptor expression, whereas males showed reduced Resistin and increased GSK3β expression. No major hypothalamic gene expression shifts were detected. Early-life exposure to a baked diet induced sex-specific metabolic alterations associated with reduced consumption of MRP-rich foods in adulthood, likely driven by peripheral metabolic signals rather than central hypothalamic regulation.

## Highlights

• Perinatal baked-diet exposure altered adult food preference in mice.
• Thermal food processing induced mice sex-specific adipose metabolic responses.
• Early-life baked-diet exposure did not prevent adult standard chow preference.
• Sensory and nutritional changes, not hypothalamic gene expression, drove preferences.

## 1 Introduction

The rise in the availability and consumption of processed foods is often associated with the shift towards increasingly sedentary lifestyles influenced by the convenience offered by industrialized food products (Bragazzi et al., 2024). The revolution in the food industry has been driven by the adoption of thermal processing techniques, which enhance both the shelf life and digestibility of foods (Featherstone, 2012). Thermal processing techniques, including pasteurization, roasting, baking, and extrusion, have facilitated the creation of a wide array of food products characterized by unique textures, colors, and aromas (de Roos, 2006; Leong & Oey, 2022). As a consequence, these methods play a pivotal role in improving sensory attributes, thereby influencing modern dietary patterns (Lemos et al., 2022; Prescott, 2015).

In recent years, increasing concerns regarding industrially processed foods have gained prominence, particularly due to their high caloric density and the extensive use of refined ingredients with low nutritional value (Levy et al., 2024). Beyond these characteristics, the intense thermal treatments employed in both industrial and domestic cooking processes can result in significant losses of thermosensitive vitamins, such as thiamine, while at the same time generating neoformed compounds (Guzman-Tello & Cheftel, 1987; Hellwig & Henle, 2014).

The reduction of thiamine content due to thermal processing has notable implications for metabolic functions. Thiamine plays an essential role in energy metabolism, particularly in carbohydrate utilization where it acts as a coenzyme in the pyruvate dehydrogenase complex and the citric acid cycle (Manzetti et al., 2014). A deficiency in thiamine has been shown to limit ATP production, reducing energy availability and potentially affecting neurological function due to the brain's high demand for glucose (Hernandez-Vazquez et al., 2016). Prolonged thiamine deficiency is associated with metabolic and neurological conditions such as beriberi and Wernicke-Korsakoff syndrome (Isenberg-Grzeda et al., 2012).

The nutritional quality of food may also be diminished by the transformation and breakdown of proteins during the formation of neoformed compounds - a key mechanism in hypotheses that seek to explain the harmful effects of so-called “ultraprocessed” foods on health (Taş et al., 2022). Many of these neoformed compounds are generated through the Maillard reaction, a non-enzymatic chemical reaction between amino acids and reducing sugars resulting in a heterogeneous group of compounds (Nogueira Silva Lima et al., 2023). Within the food matrix, this pool of Maillard Reaction Products (MRPs) encompasses multiple classes of compounds including Advanced Glycation End Products (AGEs), which have been shown to accumulate in various tissues resulting in debated influence over cellular physiology on healthy animals (Nogueira Silva Lima et al., 2024). But the Maillard reaction also contributes to the development of desirable sensory properties in foods, driven by the formation of browning pigments such as melanoidins, and volatile compounds with pleasant odor notes (Liu et al., 2022; Murata, 2021). These enhanced organoleptic characteristics have been shown to influence consumer dietary preferences, as individuals may display a preference for foods with the visually appealing brown coloration and complex aromas generated through this process (Kathuria et al., 2023; Liu et al., 2022).

Beyond the previously discussed sensory and nutritional aspects, the biomedical consequences of exposure to transformed diets of low nutritional quality from early life remain poorly defined. The intrauterine environment triggers an integrated set of adaptive responses capable of reprogramming fetal metabolism and gene expression patterns (Gluckman et al., 2007). To date, evidence linking processed food consumption to transgenerational effects has largely been derived from studies employing obesogenic dietary models (Raghavendhira et al., 2025). A recent study from our research group examined the long-term physiological effects of an isonutritional diet enriched in MRPs in mice (Nogueira Silva Lima et al., 2024). However, within this framework, the present study represents, to our knowledge, the first investigation to specifically address the impact of perinatal exposure to a thermally processed diet on offspring physiology. Dietary protocols involving thermally processed animal feed - commonly referred to as “baked chow diets” in the academic field - have been extensively explored in the literature as a means to investigate the effects of MRPs on health in animal models (Hayes et al., 2024; Mastrocola et al., 2020; van Dongen et al., 2021). Despite the potentially significant implications of thermal food processing on nutritional quality, the metabolic consequences of consuming a low-nutritional-value baked diet and its relationship with food preference behavior remain underexplored. We hypothesized that perinatal exposure to a baked diet would disrupt metabolic processes and influence food preference through metabolic and sensory-related mechanisms. To test this hypothesis, we designed an experiment which employed advanced techniques to quantify nutritional loss and Maillard reaction compounds. The primary objective of this study was to quantitatively examine the effects of perinatal exposure to thermally processed animal feed on food preference and energy metabolism in later life. The secondary objective was to study how perinatal exposure to thermally processed food may influence food intake behavior in 8-week-old, male and female C57BL/6 mice housed in a controlled, metabolic cage system.

## 2 Material and methods

### 2.1 Diet

Animal diets (A03 and A04) were obtained from SAFE Diets (Augy, France). During the reproductive period until weaning of their pups, parent animals received the A03 diet containing 23% protein; post-weaning, pups were switched to the A04 diet with 16% protein - see Section 3.1. Diets were administered in one of two forms: untransformed, standard (STD) or baked (BKD) pellets. The BKD formulations were subjected to two consecutive autoclaving cycles at 120 °C for 30 min each, followed by drying at 40 °C for two hours to reduce moisture. The diets' biochemical characteristics, presented in Table 1, reflect the variability observed across three independent production batches. Information on MRPs' stability in mice diet has been previously studied by our group (Nogueira Silva Lima et al., 2023). Nutritional and chemical analyses - including vitamin B1 (A7273–1), total lipids (AA085–3), fatty acid profile (AA25P-4), peroxide index (ID918–1), and p-anisidine value (ID920–1) - were performed by Eurofins (Nantes, France) using standardized reference methods. The glycation products, issued from the Maillard reaction, acrylamide, carboxymethyllysine (CML), furosine, and hydroxymethylfurfural (HMF), were quantified following the protocols previously established by our group (Nogueira Silva Lima et al., 2023). The colorimetric properties and volatile compound profiles of the STD and BKD diets were analyzed according to the protocol described by Troadec et al. (2023).

1.1. Animal experimentation

**Table 1:** Mice diet composition, glycation products, volatile compounds, and color formation.

Component	Unity	Diet type					
		A03			A04		
		STD	BKD	Signif.	STD	BKD	Signif.
Food composition							
Anisidine	mg/100 g	1.6 ± 0.5	3.0 ± 0.2	ns	4.4 ± 0.3	7.1 ± 1.0	ns
Lysine	mg/g DM	15.5 ± 1.0	11.8 ± 0.7	####	10.1 ± 0.6	7.0 ± 0.4	####
Monounsaturated fatty acids	g/100 g	24.7 ± 0.1	24.9 ± 0.4	ns	23.7 ± 0.7	23.6 ± 0.4	ns
Omega3	g/100 g	6.5 ± 0.0	6.4 ± 0.1	ns	5.6 ± 0.2	5.5 ± 0.1	ns
Omega6	g/100 g	2.5 ± 0.0	2.4 ± 0.0	#	1.6 ± 0.1	1.4 ± 0.1	ns
Peroxide	meq02/Kg fat	12.9 ± 1.9	10.9 ± 5.2	ns	11.8 ± 2.0	8.4 ± 0.8	ns
Polyunsaturated fatty acids	g/100 g	58.0 ± 0.2	57.4 ± 0.9	ns	56.9 ± 1.6	56.7 ± 0.8	ns
Protein	% DM	23.2 ± 0.4	23.1 ± 0.4	ns	17.1 ± 0.2	17.1 ± 0.2	ns
Saturated fatty acids	g/100 g	17.0 ± 0.0	17.4 ± 0.6	ns	19.2 ± 0.9	19.4 ± 0.4	ns
Totox	meq/Kg	27.4 ± 4.1	24.9 ± 10.4	ns	28.1 ± 4.1	23.9 ± 0.9	ns
Vitamin B1	mg/100 g	0.6 ± 0.0	0.1 ± 0.0	###	0.4 ± 0.0	0.1 ± 0.0	#
Glycation products							
Acrylamide	(μg/g DM)	<LOD	<LOD	–	<LOD	<LOD	–
Furosine	(μg/g DM)	225.8 ± 10.1	261.8 ± 18.5	####	161.3 ± 8.3	239.0 ± 17.6	####
Total dCML	(μg/g DM)	22.2 ± 1.9	98.8 ± 13.2	####	14.4 ± 1.8	77.8 ± 5.5	####
HMF	(μg/g DM)	0.1 ± 0.1	0.4 ± 0.1	####	0.2 ± 0.0	0.8 ± 0.0	####
Volatile compounds							
1-Octen-3-ol	RQ/ 2-methyl propanol	0.3 ± 0.1	0.3 ± 0.0	ns	0.5 ± 0.2	0.2 ± 0.1	##
2-Furanmethanol		0.1 ± 0.0	0.5 ± 0.2	##	0.1 ± 0.0	0.4 ± 0.2	###
3-methyl-butanal		0.5 ± 0.1	1.0 ± 0.4	#	1.0 ± 0.3	1.5 ± 0.3	ns
Hexanal		1.6 ± 0.7	1.4 ± 0.2	ns	1.4 ± 0.3	0.4 ± 0.1	##
Methyl-pyrazine		<LOD	0.1 ± 0.0	–	<LOD	0.0 ± 0.0	–
2,5-dimethyl-pyrazine		<LOD	0.1 ± 0.0	–	<LOD	0.0 ± 0.0	–
2-ethyl-5-methyl-pyrazine		<LOD	0.1 ± 0.1	–	<LOD	0.1 ± 0.0	–
3-ethyl-2,5-dimethyl-pyrazine		0.0 ± 0.0	0.1 ± 0.0	###	0.0 ± 0.0	0.0 ± 0.0	ns
Color							
a*	–	3.4 ± 0.5	7.5 ± 0.8	####	2.3 ± 0.2	6.5 ± 0.4	####
b*		21.8 ± 1.1	23.9 ± 0.9	####	18.9 ± 0.6	22.3 ± 0.9	####
C		22.0 ± 1.1	25.1 ± 1.0	####	19.1 ± 0.6	23.2 ± 0.9	####
h		81.3 ± 1.2	72.6 ± 1.7	####	83.0 ± 0.6	73.9 ± 0.6	####
L*		72.2 ± 1.5	59.6 ± 2.8	####	78.3 ± 0.7	64.6 ± 1.0	####

DM: dry matter. RQ: Relative quantification to 2-methylpropanol. LOD = Limif of detection. Total dCML = free and protein-bound CML. Totox = Total Oxidation Value. # *p* < 0.001. Statistical tests corresponded to comparisons within lines. # p ≤ 0.05; ## p ≤ 0.01; ### p ≤ 0.001; #### p ≤ 0.0001 for comparisons within A03 and A04 diet types. ns = non-significant.

**Fig. 1:**
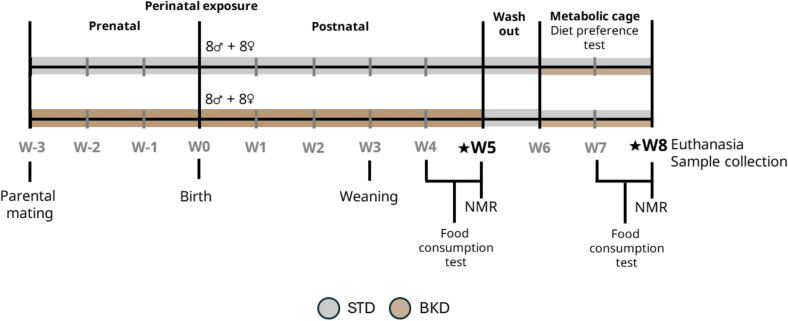
Schematic representation of experimental design. Dietary intervention was initiated during the parental reproduction period (W − 3). t birth (W0), eight male and eight female offspring per group were assigned to the same diet as their parents, either the STD or BKD diet. Pups were weaned at W3, the same period of A03 to A04 diet shift. Prior to metabolic cage testing, food intake was monitored during W4–W5, and baseline body composition was assessed at the W5 endpoint. Animals then underwent acclimation and a dietary washout period during W5–W6. The metabolic cage dietary preference test was conducted from W6–W7, with measurements of food intake, respiratory parameters, and metabolic outcomes collected at W8. (M: Males; F: Females; NMR: Nuclear magnetic resonance; STD: Standard diet; BKD: Baked diet; W: week).

All male and female, wild-type C57BL/6 mice, from homozygous wild-type founders (Charles River Laboratories, Saint-Germain-Nuelles, France), were housed under controlled conditions (21 °C, 12 h light-dark cycle, pathogen-free) at the EOPS1 Animal Facility, University of Lille. Animals were provided with standard bedding and environmental enrichment (nesting), and water and food were available ad libitum. All procedures were approved by the French Animal Ethics Committee (Protocol 232,082,019,120,215,543,800). The study followed a parallel-group, randomized design (random.org), with the individual mouse defined as the experimental unit, and allocation concealment was ensured by assigning animals to groups according to the randomization schedule without prior knowledge of group identity. Because this study investigates the effects of perinatal dietary exposure on offspring, animals necessarily originate from shared litters and parental cages. Litter effects were not modeled as a random factor, which is an inherent limitation of this type of design. While this may introduce some bias, it reflects the practical and biological constraints of studying developmental and perinatal influences, and should be borne in mind when interpreting the results. Parents were randomly assigned to one of two dietary groups: standard diet (STD) or baked diet (BKD). From the offspring, 8 males and 8 females (random pup/litter) per group were maintained on the same diet as their parents. The choice of sample size was guided by our previous studies, reporting relatively low inter-individual, within sex variability (Nogueira Silva Lima et al., 2024), as well as by logistical constraints on the number of experimental cages. Although a larger sample may have been preferable, we expected from our previous work that this design would provide sufficient statistical power to detect both main dietary effects and sex-specific responses. Dietary intervention began three weeks prior to the parents mating (W-3 – Fig. 1). Food intake of the pups was monitored at weeks 4–5 (prior to metabolic cage testing) and 7–8 (during metabolic cage testing). Body weight, fat, and lean mass were measured at weeks 5 and 8 using Nuclear Magnetic Resonance MiniSpec (Bruker, Billerica, USA). Animals were acclimatized to the NMR device prior to measurements so as to reduce handling stress, and all procedures were optimized to minimize discomfort. Following a one-week washout on STD between weeks 5–6, mice were individually housed in metabolic cages (TSE Systems, Berlin, Germany) for a two-week diet preference test (weeks 6–8), with simultaneous access to both diets. Following a two-day adaptation period, one male and one female from the STD group had failed to acclimatize to the metabolic cage environment, as evidenced by a refusal to eat, and were therefore excluded from the metabolic cage analyses, in compliance with animal welfare guidelines to minimize stress. During testing, food intake, respiratory exchange ratio, and activity were continuously recorded continuously over 24 h, with three measurements taken each hour along W6–8 (Fig. 1). Investigators were blinded to prior experimental assignments when animals were placed in metabolic cages, and cage allocation for each animal was performed randomly at the start of the experiment. Animals were monitored daily throughout the intervention and during metabolic cage housing. Health status, behavior, body weight, and food/water intake were recorded, and any signs of distress or abnormality were addressed according to institutional animal care guidelines. No early euthanasia was necessary, as no abnormalities were observed in the aforementioned parameters.

At the end of the study, after a six-hour fast (food withdrawal began at the onset of the light cycle), mice were euthanized by cervical dislocation, and blood and tissues (hypothalamus, intestines, kidneys, and gonadal white adipose tissue) were collected, flash-frozen in liquid nitrogen, and stored at −80 °C for subsequent analyses. Primary outcomes included metabolic changes, assessed by plasma markers, body composition, and tissue-specific molecular analyses. Secondary outcomes included food preference, measured by differential food intake during the diet preference test. These measures were chosen to test our hypothesis that perinatal exposure to a thermally processed diet could induce metabolic alterations and drive changes in food preference.

1.2. Biological parameters

Plasma was obtained from heparinized whole blood by centrifugation at 2000*g* for 10 min at room temperature. The supernatant plasma fraction from each individual mouse, representing a single biological replicate, was collected, aliquoted, and stored at −80 °C until biochemical analyses. Prior to analysis, plasma samples were thawed on ice. Fasting plasma glucose, total cholesterol, triglycerides, bilirubin, urea, and aspartate aminotransferase (AST) levels were quantified using an automated Spotchem EZ SP-4430 analyzer (Arkray, Minneapolis, USA), according to the manufacturer's instructions. Owing to limited plasma volume for some animals, not all biochemical parameters could be measured for every sample. These analyses, as well as those described below, were conducted blinded throughout the study.

1.3. Gene and protein panel quantifications

Gene and protein quantification in hypothalamus, ileum, colon, kidneys, and GWAT was performed using the Mouse QuantiGene Plex Expression Assay and Mouse Procarta Plex systems (ThermoFisher, Courtaboeuf, France). Owing to limited sample quantity, only the QuantiGene assay was applied to hypothalamic tissue samples. Assays followed manufacturer protocols. Gene expression was normalized to the geometric mean of endogenous controls (RPS20, RPL13a, RPS3) per organ. Protein extracts were prepared in RIPA buffer, standardized to 1 mg/mL protein quantified by Pierce BCA Kit (ThermoFisher, Courtaboeuf, France), and analyzed with ProcartaPlex Mix&Match. Plates were read on a BioRad Bio-Plex 200 system (Bio-Rad, Marnes-la-Coquette, France).

1.4. Shotgun metagenomic analysis

Cecal fecal samples were processed under anaerobic conditions prior to DNA extraction and sequencing (GenoScreen, Lille, France), following procedures adapted from Heumel (2024). Approximately 200 mg of each sample was used for genomic DNA extraction with the QIAamp Fast DNA Stool Mini Kit (QIAGEN, Hilden, Germany). Purification of DNA was performed using Agencourt AMPure XP beads (Beckman Coulter, Brea, USA). Sequencing libraries were prepared from 1 ng of purified DNA using the Nextera XT DNA Sample Prep Kit (Illumina, San Diego, USA). Libraries were sequenced on an Illumina MiSeq platform (Illumina, San Diego, USA). Raw sequencing reads were quality filtered, trimmed, and merged using Fastp v0.2, discarding reads shorter than 90 base pairs or containing more than two ambiguous bases. Read quality related data are provided in Supplemental Tables S2 and S3. To eliminate host-derived sequences, high-quality reads were mapped against the *Mus musculus* C57BL/6 J reference genome using Bowtie2. Subsequent taxonomic and functional profiling was performed with the HUMAnN3 pipeline (Beghini et al., 2021). Community structure was analyzed by principal coordinate analysis (PCoA) based on Bray-Curtis dissimilarity metrics at genus and species taxonomic levels. Differential taxonomic abundance was assessed using Linear Discriminant Analysis Effect Size (LEfSe), implemented via the R packages *lefser*, *phyloseq*, *SummarizedExperiment*, and *microbiomeMarker*. As LEfSe does not incorporate false discovery rate (FDR) correction for multiple testing, the results should be interpreted with caution due to the potential for false-positives. Functional annotation involved a two-step approach: initially, reads were mapped to the ChocoPhlan pangenome database; unmapped reads were then subjected to translated searches against the UniRef90 protein database. Gene families were annotated using UniRef90 identifiers, metabolic pathways were assigned according to MetaCyc, and enzymatic functions were classified by Enzyme Commission (EC) numbers. All abundance data were normalized to community-level relative abundances to account for differences in sequencing depth across samples.

### 2.2 Statistical analysis

Statistical analyses were performed using R (version 4.2.1) with the rstatix, ggpubr, and ggplot2 packages (R Core Team, 2022). Data were first assessed for normality using the Shapiro–Wilk test. For non-parametric comparisons, the Wilcoxon rank-sum test or the Kruskal–Wallis test was employed, as appropriate, followed by Dunn's post hoc multiple-comparison test with Bonferroni adjustment. Data are presented as means ± standard error of the mean (SEM) in bar plots, or as medians with interquartile ranges in box plots. Statistical significance was determined using an overall α level of 0.05. Specific statistical methods used for each analysis are indicated in the corresponding figure legends.

## 3 Results and discussion

1.5. Characteristics of diets and effect of baking

Two diets were used in this study with differing protein levels in order to meet the animals' macronutrient needs at different stages of development, namely: A03 (23 g/100 g food) during reproduction and early postnatal weeks, and A04 (17 g/100 g food) from week 3 onwards. Thermal treatment did not significantly alter the total protein content in either diet (Table 1). The A03 STD protein content was equivalent to 23.2 ± 0.4%, while A03 BKD total protein was 23.1 ± 0.4%. Similarly, A04 STD total protein was equivalent to 17.1 ± 0.2% and was 17.1 ± 0.3% in A04 BKD. Although no substantial changes were noticeable in total protein content, the thermal treatment resulted in a significant (*p* < 0.0001) reduction in lysine residues (Table 1). A 24–30% decrease in lysine content was observed between A03 STD (15.5 ± 1.0 mg/g dry matter (DM)) and A03 BKD diets (11.8 ± 0.7 mg/g DM) and between A04 STD (10.1 ± 0.6 mg/g DM) and A04 BKD diets (7.1 ± 0.4 mg/g DM). This decrease in lysine concentration was partially explained by the formation of lysine-born MRPs, including early glycation markers like fructose-lysine (here reported as its acid-hydrolysis derivative furosine), as well as advanced glycation end-products (AGEs) such as CML and HMF (Table 1). Furosine concentrations increased from 225.8 ± 10.1 μg/g DM in A03 STD to 261.8 ± 18.5 μg/g DM in A03 BKD, and from 161.3 ± 8.3 μg/g DM to 238.9 ± 17.6 μg/g DM in A04 STD and A04 BKD, respectively (*p* < 0.0001). The CML content in BKD diets was approximately 4.5 times higher than in non-baked diets, rising from 22.2 ± 1.9 μg/g DM in A03 STD to 98.8 ± 13.2 μg/g DM in A03 BKD, and 14.4 ± 1.8 μg/g DM in A04 STD to 77.8 ± 5.5 μg/g DM in A04 BKD (*p* < 0.0001). A similarly pronounced increase (3 to 5-fold) was observed for HMF (0.1 ± 0.1 μg/g DM in A03 STD to 0.4 ± 0.1 μg/g DM in A03 BKD; 0.2 ± 0.0 μg/g DM to 0.8 ± 0.0 μg/g DM in A04 STD and A04 BKD, respectively (*p* < 0.0001)). Although direct comparisons with other studies are complicated by differences in food composition and heating methods, baking animal diets has been reported by others to increase glycation products like CML and HMF by roughly fivefold (Anton et al., 2012; van Dongen et al., 2021).

Glycation also affects amino acids other than lysine, such as arginine, serine and cysteine, producing diverse MRPs that promote browning and the formation of volatiles (Fadel et al., 2023). In our study, thermal treatment intensified the Maillard reaction, leading to a change in aroma profiles. The 2-furanmethanol content (burnt note) increased significantly in both diets upon baking, as did the 3-methylbutanal content (malty, roasted note) in diet A03 (Table 1). In addition, the detection of pyrazines (methyl pyrazine, 2,5-dimethyl pyrazine, 2-ethyl-5-methyl pyrazine) in baked formulations probably reinforced the burnt and toasted aroma. Conversely, volatile compounds (1-octen-3-ol; hexanal) resulting from lipid oxidation tended to decrease in baked formulations, which may also have affected the overall aroma of the formulations. The Maillard reaction also contributes to food browning via melanoidin formation. Using colorimetry, we assessed surface color changes in modified diets. Heated BKD diets exhibited a darker appearance, marked by a significant decrease in lightness value (L*) and altered color parameters. The L* decreased after thermal processing from 72.2 ± 1.5 to 59.6 ± 2.8 in the A03 diet, and from 78.3 ± 0.7 to 64.6 ± 1.0 in the A04 diet.

Thermal processing not only altered the diets' amino acid profile, but also their lipid, and vitamin profiles, notably reducing heat-sensitive Omega-6 fatty acids and vitamin B1 (thiamine). A slight but significant decrease in Omega-6 concentrations was observed in the A03 diet, (2.5 ± 0.0 g/100 g in A03 STD to 2.4 ± 0.0 g/100 g in A03 BKD (*p* < 0.05). A similar, but not significant, trend was noted in the A04 diet with Omega-6 levels (Table 1). A more pronounced reduction was observed in vitamin B1 concentrations, with thermal treatment leading to a decrease in both A03 and A04 diets. Specifically, the vitamin B1 content in A03 dropped from 0.6 ± 0.0 mg/100 g in A03 STD diet to 0.1 ± 0.0 mg/100 g in the A03 BKD diet (*p* < 0.001), while in A04 it decreased from 0.4 ± 0.0 mg/100 g in A04 STD diet to 0.1 ± 0.0 mg/100 g in the BKD diet (*p* = 0.02).

### 3.1 Provision of the baked diet neither increased total dietary intake nor induced food preference, independent of whether animals had access to a choice between diets

**Fig. 2:**
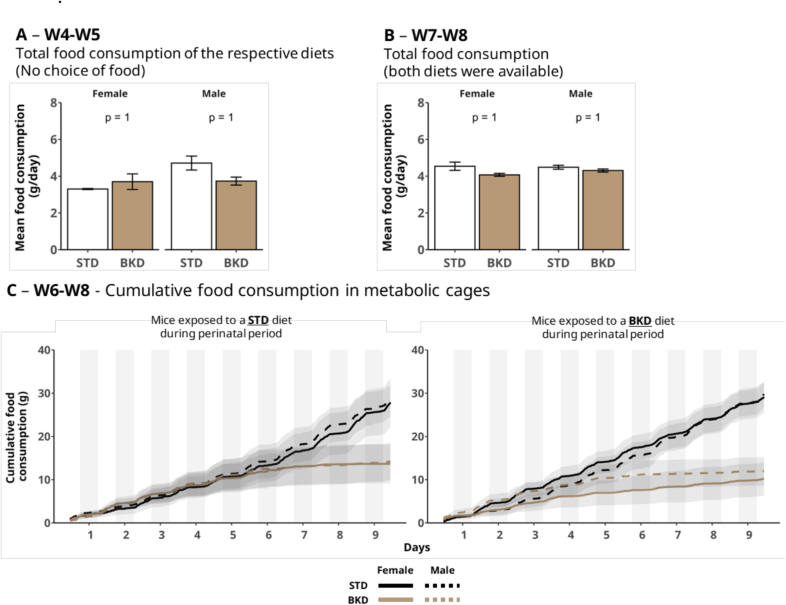
**Consumption of STD and BKD diets.** (A) Mean food intake during W4–W5 under single-diet conditions (STD or BKD). (B) Mean food intake during W7–W8 in metabolic cages under a two-choice paradigm (STD vs. BKD). X-axis labels indicate the diet to which mice were exposed during the perinatal period (W − 3–W5). Food intake was analyzed separately within each sex to account for inherent sex-specific differences in consumption (Wilcoxon test, α = 0.05). Bars represent mean ± SEM (*n* = 8 sex/group). (C) Temporal profile of cumulative food intake in metabolic cages from W6–W8. Vertical shaded areas denote light-phase periods. Lines represent mean cumulative food intake ± SEM (shaded area). Female/STD *n* = 7: Male/STD n = 7; Female/BKD n = 8: Male/BKD *n* = 8. Multiple-group comparisons were performed using the Kruskal–Wallis test (*p* > 0.05).

There were no differences in total food intake between STD and BKD groups as measured in W5 (Fig. 2A). Observed food consumption at this time point ranged from 3.7 ± 0.2 to 4.7 ± 0.4 g/day for males and from 3.3 ± 0.0 to 3.7 ± 0.4 g/day for females. Neither was there a difference in food intake between STD and BKD groups when a choice of diet was available in metabolic cages during W7–W8 (Fig. 2B). The overall food intake between W7 and W8 was not significantly different between males (4.3 ± 0.5 to 4.5 ± 0.6 g/day) and females (4.1 ± 0.5 to 4.5 ± 1.3 g/day) in the metabolic cages. Several studies comparing baked and control diets have reported a similar lack of significant differences in total consumption (Anton et al., 2012; Merhi et al., 2025; van Dongen et al., 2021). Our findings demonstrate that food intake per se remains unchanged between animals exposed perinatally to, and subsequently reared on, either a STD or BKD diet, regardless of sex or age. Additionally, no significant differences were observed in total food intake when animals were simultaneously offered both diets.

To our knowledge, this is the first study to investigate the effects of perinatal exposure to a low-thiamine, MRP-rich baked diet on food intake in later life, as well as subsequent metabolic and behavioral outcomes. Fig. 2A and B show that perinatal exposure to a particular diet did not affect overall food intake upon weaning (*p* = 1); however, Fig. 2C shows that, when given a choice between the two diets in a metabolic cage, both groups tended to prefer the STD diet over the BKD diet, indicating that early-life exposure to the BKD diet may not have substantially affected dietary preference. Initially, both diets were consumed equally, but intake of the BKD diet declined over time, while STD diet consumption increased. This shift occurred consistently across sexes and independent of early-life diet exposure. Interestingly, mice perinatally exposed to and weaned upon the BKD diet shifted to higher STD consumption within only three days, while those raised on the STD diet took about six days to show a similar change. Prior to testing in metabolic cages, all animals underwent a one-week washout on the STD diet. These results therefore suggest the possibility that perinatal and early-life exposure to BKD may be retained as a form of early dietary memory and could contribute to a later avoidance of BKD-associated foods when given a choice. This hypothesis warrants further investigation, particularly into the sensory-driven mechanisms underlying food preference and aversion. The one-week washout period on a standard diet prior to the preference test was implemented to reduce potential acute dietary effects; however, its duration was arbitrarily chosen, as this study is the first to investigate sensory responses to baked diets in mice. While this approach allowed for standardized testing conditions, it may have partially attenuated early-life programming effects and introduced carry-over or metabolic adaptation biases. Future studies could explore the effect of washout periods on perinatal programming in order to characterize the influence of this procedure, and indeed its necessity.

It is essential, however, to consider other factors that may have influenced the observed eating behavior. Compared to the STD diet, the BKD diet showed higher MRP levels, reduced lysine availability, and lower vitamin B1 content due to thermal processing. These changes raise the possibility that food preference was shaped by both the sensory properties of MRPs and the decreased nutritional quality of the BKD diet. From a sensory standpoint, MRPs are known to enhance color, taste, and aroma, potentially increasing food appetence (Starowicz & Zieliński, 2019; Sun et al., 2022). However, the specific influence of flavor-related MRPs on food preference remains underexplored. In our study, the BKD formulation did not appear to increase palatability for mice, which may reflect diet-specific factors such as excessive MRP formation, or other baking-related changes. Negative sensory traits, such as bitterness or rancidity, may also influence feeding behavior, and certain MRPs may exceed sensory thresholds, potentially leading to unpleasant flavor profiles (Baigrie, 2003; Li et al., 2023). As a case in point, compounds like 1-octen-3-ol, pyrazines, and hexanal are known to impart off-flavors such as bitterness, earthiness, or rancidity, depending on the food matrix (Qiu et al., 2024; Starowicz & Zieliński, 2019; Zhao et al., 2021). For animals like mice, their olfactory sensitivity may play a role in reducing their overall consumption of thermally processed foods (Zou et al., 2015).

The micronutrient content of the diets may also have influenced feeding behavior, as described by the well-established concept of ‘nutritional wisdom’ whereby mammals instinctively adjust food intake according to their nutritional needs (Brunstrom & Schatzker, 2022). This theory proposes that animals learn to link a food's sensorial properties with its nutritional value, adjusting their diet based on this association (Ackroff et al., 2007). A seminal study conducted on male and female rats provided evidence that thiamine-deficient animals showed a preference for a diet supplemented with vitamin B1 when given a choice (Rozin et al., 1964). In this context, a lack of key nutrients such as vitamins, lost during thermal processing in our study, may have participated in the animals' preference for the STD diet. This finding supports the idea that the physiological effects of nutrient intake, combined with sensory cues, play a critical role in shaping food choices. Hence, MRPs appeared to act as a sensory cue of reduced food quality, especially for animals perinatally exposed to and weaned on the BKD diet who shifted more rapidly to the STD diet. The observed preference of both groups for the STD diet over the BKD diet is consistent with nutritional wisdom, perhaps through a combined influence of nutritional and sensory factors - including differences in thiamine and lysine content, lipid oxidation, or off-flavors from baking - but our current data do not allow us to attribute this effect to either the reduced nutritional value or the MRPs that characterized the BKD diet.

### 3.2 Compulsory BKD diet intake results in lower lean mass in male mice

**Fig. 3:**
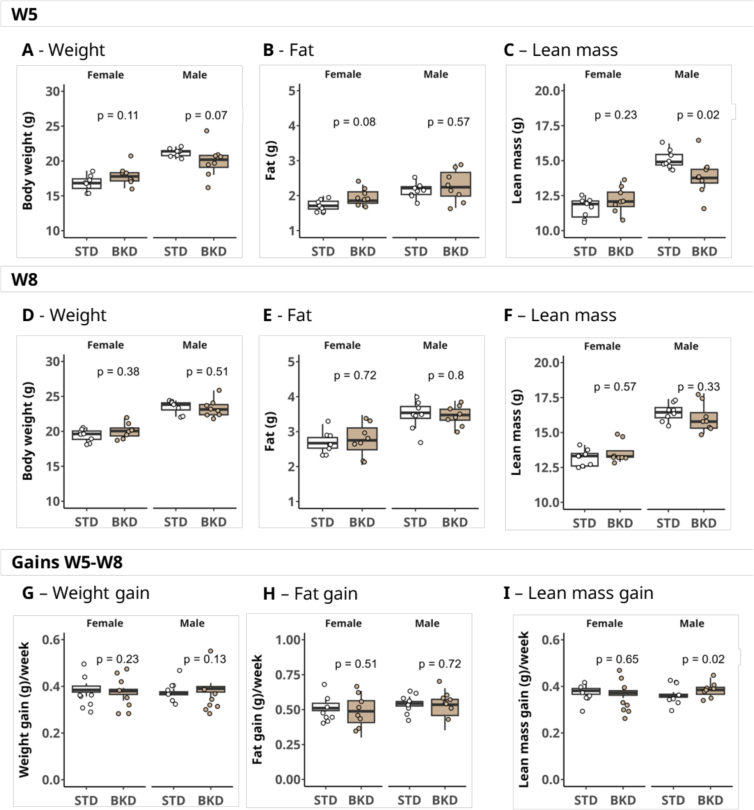
Effects of perinatal exposure to the BKD diet on body composition. Perinatal exposure to the BKD diet resulted in reduced lean mass in male mice at W5, which was no longer observed at W8. Prior to metabolic cage experiments (W5), body weight (A), fat mass (B), and lean mass (C) were assessed. Following metabolic cage testing (W8 endpoint), body weight (D), fat mass (E), and lean mass (F) were measured. Changes in body weight (G), fat mass (H), and lean mass (I) from W5 to W8 are expressed as gains (g/week). Data are presented as mean ± SEM (n = 8 per sex/group. Wilcoxon test, α = 0.05).

Body composition measurements were conducted at W5 and at W8 time points. Among animals of the same diet, age, and sex, there were no significant differences in body weight, fat, and lean mass values (median (g) [IQR]) at W5 (Fig. 3A, 2B, C). However, a statistically significant lower lean mass was observed in male mice perinatally exposed to and weaned upon the BKD diet (13.8 g [13.4: 14.4]) compared with those on the STD diet (14.9 g [14.9: 15.4]; *p* = 0.02) (Fig. 3C). This effect was not observed among female animals and was no longer evident at W8 (Fig. 3G), as BKD-fed males exhibited a significantly greater (*p* = 0.02) lean mass gain (0.4 g/week [0.36: 0.39]) compared with STD-fed males (0.4 g/week [0.35, 0.36]) (Fig. 3I) during this 3-week period. No differences were detected in calorimetric measures and respiratory exchange during testing in the metabolic cages (Supplementary fig. S1A, S1B). A comparable absence of differences in body weight and fat mass ratios following intake of a baked chow diet have been reported by others in male mice (9 weeks of age) and rats (12 weeks of age), in line with our findings (Hayes et al., 2024; van Dongen et al., 2021).

**Fig. 4:**
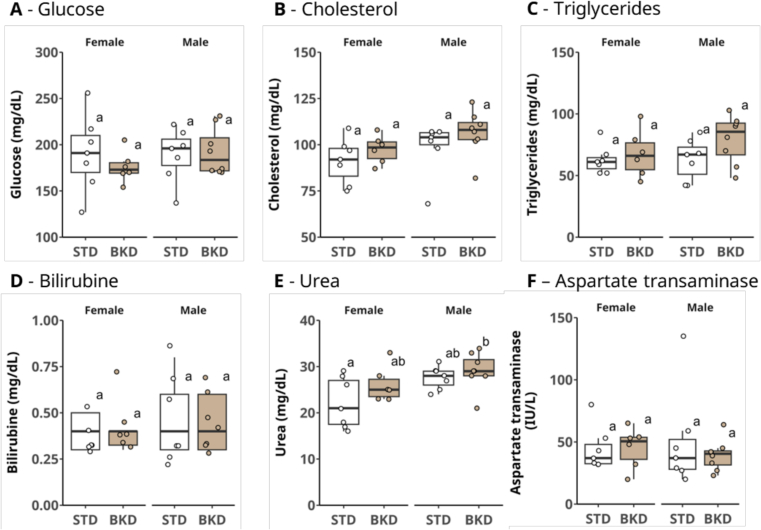
**Blood biochemical parameters.** Blood concentrations of (A) glucose (mg/dL), (B) cholesterol (mg/dL), (C) triglycerides (mg/dL), (D) bilirubin (mg/dL), (E) urea (mg/dL), and (F) aspartate aminotransferase (IU/L). Due to limited plasma volume for some animals, not all biochemical parameters could be measured for every sample. Female/STD n = 7: Male/STD n = 7; Female/BKD *n* = 6: Male/BKD n = 8. Different letters indicate statistically significant differences among groups within each parameter (Kruskal–Wallis test followed by Dunn's post hoc multiple-comparison test; α = 0.05).

An assessment of biomarkers related to glucose, lipid, and protein metabolism of animals at week 8 is illustrated in Fig. 4. With the exception of circulating urea concentrations, there were no differences attributable to either diet or sex among the groups. Although differences were small, urea concentrations (median (mg/dL) [IQR]) were higher in male mice raised on the BKD diet (29.0 [28.0: 31.5]) compared with female mice on the STD diet (21.0 [17.5: 27.0]) (*p =* 0.048; Fig. 4E).

Protein metabolism is dependent on diet quality (Kim et al., 2018), and preservation of muscle mass requires both essential amino acids and sufficient vitamins, both in humans (Mithal et al., 2013) and rodents (Ersoy et al., 2024). In our study, male mice raised on the BKD diet had lower lean mass and higher circulating urea concentrations, suggesting increased muscle catabolism. Glycated Bovine Serum Albumin (BSA) has been shown to impair muscle homeostasis via insulin resistance, endoplasmic reticulum stress, and PERK/FOXO1 activation (Du et al., 2023). In addition, the significant loss of vitamin B1 from thermal processing in our BKD diet cannot be overlooked. The BKD diet's low vitamin B1 content (0.1 mg/100 g vs. 0.3 mg/100 g recommended; Nutrition, 1995) may further disrupt glucose and amino acid metabolism, and B1 deficiency has been shown to promote muscle wasting in C57BL/6 mice (Liu et al., 2014), in line with our observations.

### 3.3 Gonadal fat pads reveal a sex dimorphic response to the BKD diet

**Fig. 5:**
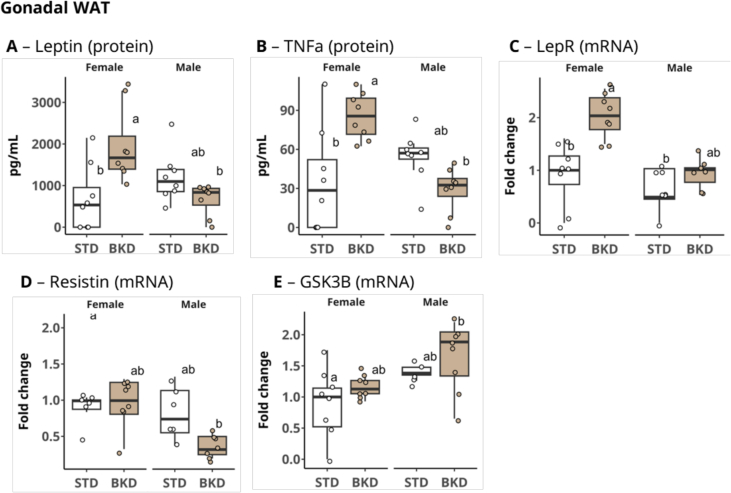
Gonadal white adipose tissue (GWAT) expression of inflammatory and metabolic markers. Protein expression of (A) leptin and (B) TNFα, and gene expression of (C) *LepR*, (D) *Resistin*, and (E) *GSK3B* in GWAT. Different letters indicate statistically significant differences among groups within each biomarker (Kruskal–Wallis rank test followed by Dunn's post hoc multiple-comparison test; α = 0.05). Data are presented as mean ± SEM (n = 8 sex/group).

Quantitative analysis revealed that Leptin (LEP) and Tumor Necrosis Factor Alpha (TNFα) secretion were significantly higher by 2.8-fold (*p* = 0.009) and 2.4-fold (*p* = 0.01), respectively, in the gonadal adipose tissue of female mice raised on the BKD diet compared with females raised on the STD diet (Fig. 5A, B). Such differences occurred in the LEP and TNF-α protein levels suggesting transcriptional regulation of the corresponding coding genes. While *LepR* expression was found to be 2-fold higher among the female mice raised on the BKD diet (*p* = 0.01) (Fig. 5C), male mice were not different in this regard. In male mice, comparing both diets, no significant differences were observed in glucose homeostasis–related signaling genes, despite a tendency for a reduction in *Resistin* expression accompanied by increased expression of glycogen synthase kinase 3 beta *(GSK3β)*. (Fig. 5D, E).

Elevated LEP and inflammatory protein (TNFα) in female gonadal white adipose tissue, alongside reduced *Resistin* expression in males, suggest a complex interplay of metabolic, hormonal, and immune factors shape responses to diet (Friedman, 2019; Yeung & Tadi, 2024). Female mice have been suggested to be more sensitive to metabolic and hormonal stressors, perhaps explaining their stronger LEP response (Omotola et al., 2019). This sexual dimorphism in energy regulation, driven by estrogen and related pathways, likely underlies differences in LEP levels and adipose tissue, as has been widely documented (De Jesus & Henry, 2023). Elevated *Resistin* is associated with insulin resistance and inflammation (Tripathi et al., 2020). In males, the trend towards reduced *Resistin* expression, alongside increased GSK3β expression, may indicate altered glucose homeostasis in the BKD group relative to the STD group. Although lower *Resistin* may reduce inflammation, heightened *GSK3β* promotes insulin signaling, enhancing protein degradation and potentially leading to muscle atrophy (Wang et al., 2006). A recent study by Merhi et al. (2025) found that perinatal exposure (pregnancy and lactation) to a baked diet significantly impacted long-term adiposity, hormonal balance, and glucose metabolism in CD1 mice. Despite methodological differences, their results broadly align with our findings, underlining the effects of baked diets on body composition and metabolic regulation (Merhi et al., 2025).

While our study revealed sex-specific differences in metabolic responses, their underlying mechanisms – whether due to perinatal MRP exposure or vitamin B1 deficiency - seem to converge. A key finding from pertinent literature is that MRP-enriched diets consistently lead to adipose tissue inflammation through diverse pathways. The accumulation of protein-bound CML in adipose tissue has been correlated with increased cytokine expression in a process mediated by the RAGE receptor during adipogenesis, both in vivo and in vitro (Gaens et al., 2014). Through immunocytochemical staining, Gaens et al. (2014) demonstrated that CML accumulates in subcutaneous adipose tissue and is coincident with the cellular localization of RAGE. Adipose tissue inflammation and elevated LEP levels are also linked, arising from either increased adiposity or compensatory hyperleptinemia due to LEP resistance, as shown in rodent models (Pretz et al., 2021). In addition, LEP levels have been consistently correlated with higher expression of TNFα and RAGE, and concentrations of circulating MRPs (Uribarri et al., 2015).

In this study, there were only a few sex-specific and relatively small differences in adipose biomarkers between animals raised on the STD or BKD diets, suggesting that the observed changes in gene and protein expression are not solely explained by a difference in intake of MRPs. The reduction in thiamine levels caused by thermal processing of the diet may also be a contributing factor. Thiamine deficiency can mimic leptin's effects by activating AMPK signaling in both the brain and peripheral tissues (e.g. muscles), potentially leading to an anorexigenic state (Liu et al., 2014). It is hypothesized that vitamin B1 deficiency may influence LEP signaling by increasing LEP/receptor expression or enhancing leptin's intracellular pathways (Liu et al., 2014). Vitamin B1 deficiency is also known to impair glucose metabolism, a process regulated by GSK3β. While these metabolic mechanisms may contribute to the dietary choices observed in our metabolic cage experiments, we acknowledge that sensory/palatability differences between the BKD and STD diets were not explicitly controlled for in the current study. Overall, our findings suggest that dietary thiamine deficiency and LEP signaling dysfunction, potentially interacting with sensory cues, may have contributed to animals' preference for the nutritionally more complete STD diet.

### 3.4 Consumption of the BKD diet did not influence hypothalamic appetite control

**Fig. 6:**
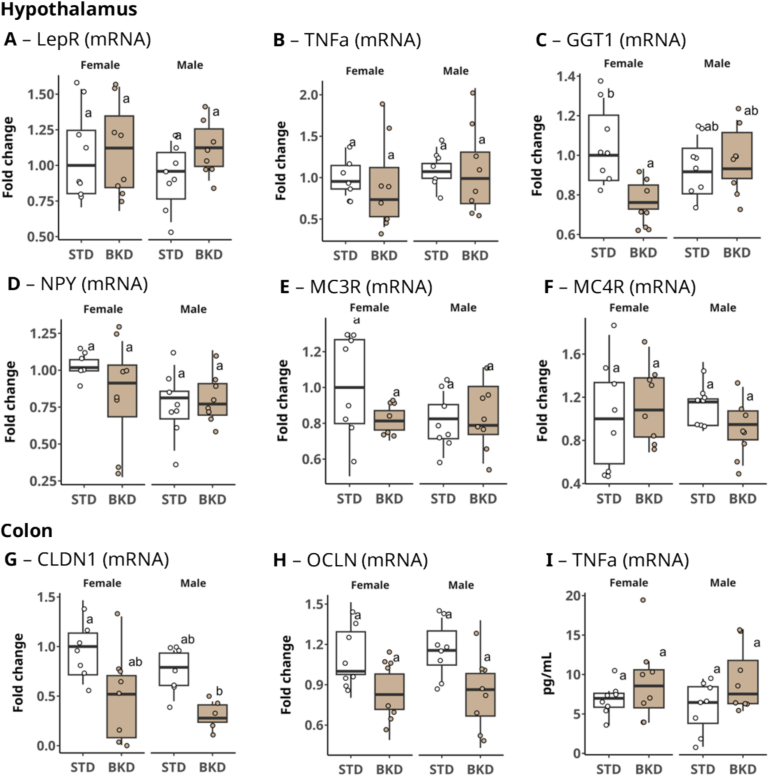
Hypothalamic and colonic expression of metabolic and inflammatory markers. Hypothalamic mRNA expression of (A) *LepR*, (B) *TNFα*, (C) *GGT1*, (D) *NPY*, (E) *MC3R*, and (F) *MC4R*. Colonic expression of (G) *CLDN1* (mRNA), (H) *OCLN* (mRNA), and (I) *TNFα* (protein). Different letters indicate statistically significant differences among groups within each tissue and biomarker (Kruskal–Wallis rank test followed by Dunn's post hoc multiple-comparison test; α = 0.05). Data are presented as mean ± SEM (n = 8 sex/group).

Given the observed increase in LEP secretion in the GWAT of female mice, we subsequently investigated the associated pathways related to the action of this hormone in the hypothalamic region. No differences in the expression of LEP-related genes were detected within the hypothalamus, including *LepR* (Fig. 6A) and pro-inflammatory cytokines such as *TNFα* (Fig. 6B). But distinct signs of disruption to hypothalamic homeostasis were observed in female mice raised on the BKD diet, notably a significant 1.5-fold decrease in the expression of Gamma-Glutamyltransferase 1 (*GGT1)* (*p* = 0.019, Fig. 6C), indicating an imbalanced redox state. We also quantified the hypothalamic expression of genes associated with satiety, appetite, and food reward. Key appetite regulators, including Neuropeptide Y (*NPY)*, Melanocortin 3 Receptor (*MC3R)*, and Melanocortin 4 Receptor (*MC4R)*, were unaffected by the overall food intake and showed no significant sex-based differences in their expression levels (Fig. 6D, E, F).

Our study indicates that the BKD diet did not affect appetite regulation or induce hyperphagic behavior, which aligns with previously reports (Hayes et al., 2024; van Dongen et al., 2021). Understanding how genes like *NPY* (appetite-stimulating), *MC3R*, and *MC4R* (appetite-suppressing) interact sheds light on how a diet rich in MRPs, and deficient in essential nutrients, may impact feeding behavior (Bedenbaugh et al., 2022; You et al., 2016). Maintaining energy balance relies on the equilibrium of these signaling pathways. This highlights that observed metabolic differences stem from differences in the nutritional quality of food rather than the quantity consumed.

Nor did the BKD diet have a significant impact on the gastrointestinal system. The intestinal epithelial barrier remained largely intact; colon Claudin-1 *(CLDN1)* and Occludin *(OCLD)* gene expression showed non-significant differences, with a slight downregulation trend among the BKD animals (Fig. 6G, H). But when samples were compared on the basis of diet alone (both sexes pooled – data not shown), this downregulation was statistically significant for both *CLDN1* (*p* = 0.006) and *OCLD* (*p* = 0.001), which accords with recent results published by Snelson et al. (2021). Crucially, there was no significant local inflammation or cell cycle disruption, indicated by stable colonic *TNFα* levels (Fig. 6I). This lack of inflammation was also seen in the ileum and kidneys (Supplementary fig. S2).

The relationship between LEP, *CLDN1*, and *OCLN* is vital for understanding how metabolic imbalances impact peripheral and central barrier functions, driving inflammatory and metabolic diseases. A compromised colonic barrier could permit bacterial translocation, activating immune and neuroinflammatory pathways. While we observed only a trend in gene downregulation, MRP-rich diets are been reported to impair tight junction integrity, leading to poor intestinal epithelial health and morphological changes (Phuong-Nguyen et al., 2023).

In addition, a baked diet has been shown to reduce cecal microbiota diversity and increased harmful bacteria in Sprague-Dawley rats, alongside structural colonocyte changes and decreased tight junction proteins, indicating compromised intestinal permeability due to high levels of dietary AGEs (Qu et al., 2017). Notably, thiamine is essential for barrier function via redox balance and energy homeostasis (Ma et al., 2022), and thiamine deficiency has been shown to decrease tight-junction expression in various mouse tissues, including the colon (Beauchesne et al., 2009).

### 3.5 Gut microbiota is resilient to lower nutritional quality and higher MRP intake

**Fig. 7:**
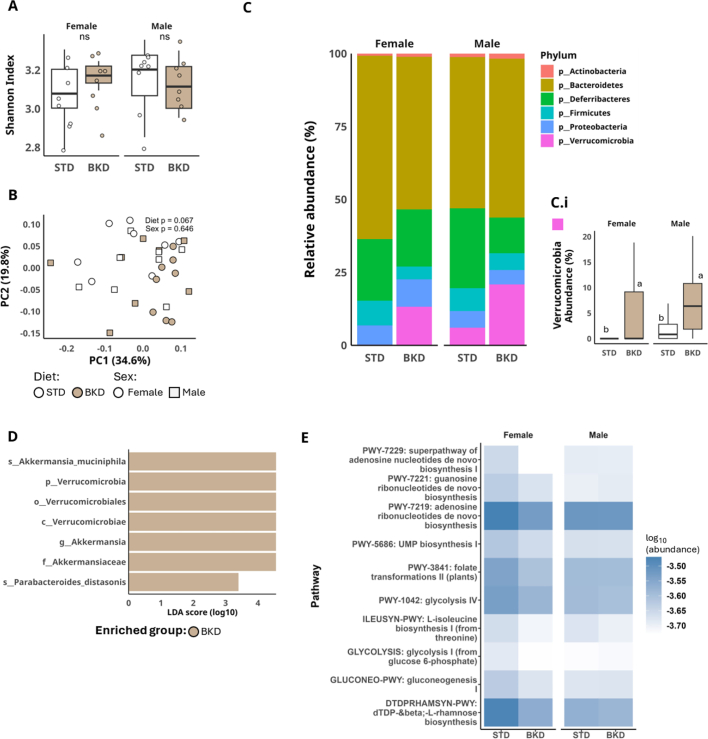
**Cecal microbiota shotgun metagenomic analysis.** (A) α-diversity estimated using the Shannon index. (B) Principal coordinates analysis (PCoA) based on Bray–Curtis dissimilarity, with each point representing an individual cecal sample (R^2^_diet_ = 0.008, R^2^_sex_ = 0). (C) Relative abundance of bacterial phyla stratified by diet and sex; (C.i) boxplot of *Verrucomicrobia* phylum relative abundance. (D) Linear discriminant analysis effect size (LEfSe) identifying taxa discriminating experimental groups (α = 0.05). (E) Functional metabolic pathway abundance (log₁₀), inferred from shotgun metagenomic data, in male and female mice exposed to STD or BKD diets. Taxonomic profiling was performed using MetaPhlAn, and functional pathway annotation was conducted using HUMAnN. Different letters indicate statistically significant differences among groups within each panel (Kruskal–Wallis test followed by Dunn's post hoc multiple-comparison test). *ns* = non-significant differences. Data are presented as mean ± SEM (n = 8 sex/group).

Metagenomic shotgun sequencing of male and female mice on STD or BKD diets revealed no significant changes in alpha-diversity of gut microbiota (Shannon Index), indicating that within-sample bacterial diversity was largely unaffected by diet (Fig. 7A). However, beta-diversity analysis (PCoA) showed a trend towards diet-dependent clustering (*p* = 0.07; Fig. 7B), suggesting that the BKD diet altered overall microbial community composition. These analyses were performed using a read-based approach via HUMAnN3, which enables comprehensive taxonomic and functional profiling directly from sequencing readings, but does not provide genome-resolved metagenomes (MAGs). This approach has inherent limitations, including the lack of genome-level resolution and the potential for unmapped reads. In addition, the limited sample size and sequencing depth reduce statistical power. Consequently, no robust differences were detectable under the current conditions, and the analyses should be considered as exploratory. Overall, these findings align well with previous studies on similar baked diets (van Dongen et al., 2021) and suggest that early-life dietary exposure can leave lasting microbial signatures.

The most prominent taxonomic shift, observed in both sexes, was the significant enrichment of the phylum Verrucomicrobia, with *Akkermansia muciniphila* identified as the predominant species (Fig. 7C, D). The LEfSe analysis confirmed these taxa as discriminative features of the BKD diet (LDA cutoff = 2; *p* ≤ 0.05). The rise of *A. muciniphila*, a mucin-degrading bacterium associated with gut barrier function and metabolic health (Plovier & Cani, 2017), is a striking finding, potentially reflecting an adaptation to the BKD diet. Our group has previously reported similar impacts on other mucin-degrading taxa with increased consumption of AGEs (Nogueira Silva Lima et al., 2024).

Despite the observed taxonomic shifts, predicted microbial metabolic function, as inferred from pathway abundance, showed no statistically significant differences between dietary or sex groups (Fig. 7E). Although differences were noted in pathways such as purine and pyrimidine biosynthesis, glycolysis/gluconeogenesis, and dTDP-4β,6-deoxy-α-D-mannose biosynthesis (DTDRPHAMSYN-PWY), none was statistically significant. This suggests that the functional metabolic potential of the microbial communities remained stable across all experimental conditions and throughout the study duration.

Sex-specific microbial responses to the BKD diet were evident, with both females and males showing increased Verrucomicrobia but varying magnitudes of change and involvement of specific taxa. These differences likely stem from sex hormones, immune modulation, or metabolic demands, highlighting biological distinctions in diet processing and host-microbiota interactions. Understanding these sex-dependent effects is crucial for targeted dietary interventions and may explain observed physiological differences between male and female mice, potentially via gut-brain axis signaling and hormonal resilience.

## 4 Conclusions

This study demonstrates that perinatal exposure to thermally processed foods -characterized by elevated levels of MRPs and reduced nutritional quality, notably lower lysine and thiamine content - induces subtle yet significant alterations in mouse physiology. Sex-dependent effects were observed on body composition, endocrine regulation, and feeding behavior. Specifically, males raised on the baked diet exhibited reduced lean mass at weaning, whereas females displayed increased adiposity-associated inflammatory markers in gonadal white adipose tissue. Behavioral analyses indicated that feeding preferences were influenced by sensory and/or nutritional factors but could not be attributed solely to sensory memory formation. In addition, the dietary intervention altered gut microbiota composition, with a notable increase in *Akkermansia muciniphila*. Although this study demonstrates the combined influence of nutrient loss and exposure to an MRP-rich diet during perinatal and early life on metabolism and food preference, disentangling the independent contributions of MRPs and nutrient degradation remains challenging. Nevertheless, our findings underscore the importance of considering micronutrient loss – here exemplified by thiamine - and sex-specific responses to thermally processed foods, which may be particularly relevant in the context of the rising consumption of ultra-processed diets. Other sensory features, such as bitterness, may also have contributed to our findings. Future studies addressing sensory perception thresholds, the intergenerational influence of feeding habits, and the long-term consequences of early-life micronutrient deprivation on food intake will be essential to further clarify these mechanisms.

## CRediT authorship contribution statement

**M.T. Nogueira Silva Lima:** Writing – review & editing, Writing – original draft, Visualization, Validation, Project administration, Methodology, Investigation, Formal analysis, Data curation, Conceptualization. **C. Delayre-Orthez:** Writing – review & editing, Methodology, Conceptualization. **M. Howsam:** Writing – review & editing, Validation, Methodology. **P. Jacolot:** Writing – review & editing, Visualization, Validation, Methodology, Investigation. **C. Niquet-Léridon:** Writing – review & editing, Visualization, Validation, Methodology, Investigation, Formal analysis. **A. Okwieka:** Writing – review & editing, Methodology, Formal analysis. **P.M. Anton:** Writing – review & editing, Conceptualization. **M. Perot:** Writing – review & editing. **A. Ghinet:** Writing – review & editing. **C. Jouquand:** Writing – review & editing, Methodology, Investigation, Formal analysis. **C. Fradin:** Writing – review & editing. **E. Boulanger:** Writing – review & editing, Funding acquisition. **S. Jaisson:** Writing – review & editing, Conceptualization. **P. Gillery:** Writing – review & editing, Supervision, Project administration, Funding acquisition, Conceptualization. **F.J. Tessier:** Writing – review & editing, Supervision, Project administration, Methodology, Funding acquisition, Conceptualization.

## Funding

This work was funded by Agence Nationale de la Recherche (ANR) [34–0013CE].

## Declaration of competing interest

The authors state that they have no conflicts of interest, whether financial or personal, that could be perceived as influencing this research.

## Data Availability

Data will be made available on request.

## Appendix A Supplementary data

Average energy expenditure measured from week 6 to week 8. Inflammation profiling in the ileum and kidneys. Summary of DNA Sequencing Data. Quality Control and Preprocessing Summary for 35 Sequencing Libraries Image 1
