# Supplementary material for: Perinatal consumption of a thermally processed diet alters feeding behavior and has sex-dependent effects upon metabolism in later life in mice
Source: Food Chem (Oxf). 2026 Feb 19;12:100375. doi: 10.1016/j.fochms.2026.100375 (PMC13080651; doi:10.1016/j.fochms.2026.100375)
Supplement: Supplementary file 1 — Average energy expenditure measured from week 6 to week 8. Inflammation profiling in the ileum and kidneys. Summary of DNA Sequencing Data. Quality Control and Preprocessing Summary for 35 Sequencing Libraries [file mmc1.docx]

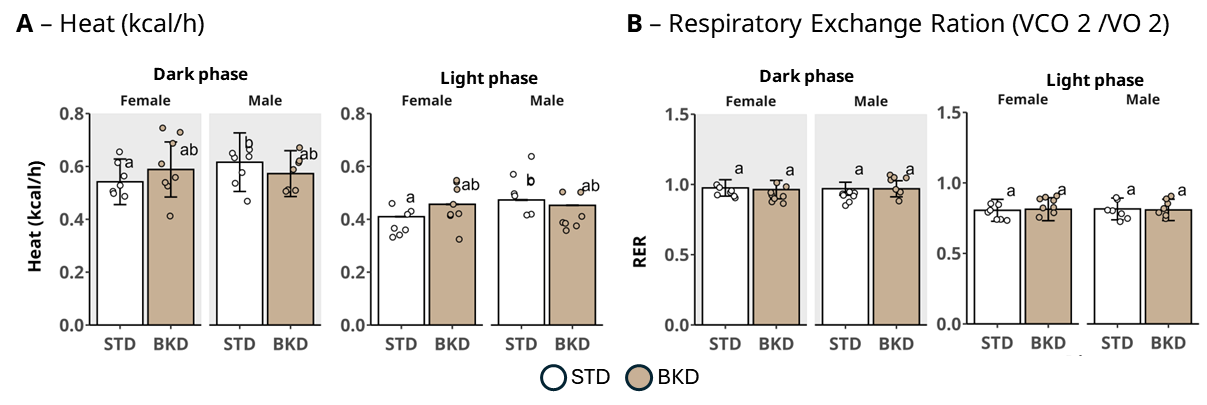


**Figure S1.** Average energy expenditure measured from week 6 to week 8. **(A)** Nocturnal and diurnal heat production (kcal/h). **(B)** Respiratory Exchange Ratio (RER; VCO₂/VO₂). Bars represent mean ± SEM (n = 8 sex/group). Different letters indicate statistically significant differences among groups within each organ (Kruskal–Wallis test followed by Dunn’s post hoc test for multiple comparisons, α = 0.05).


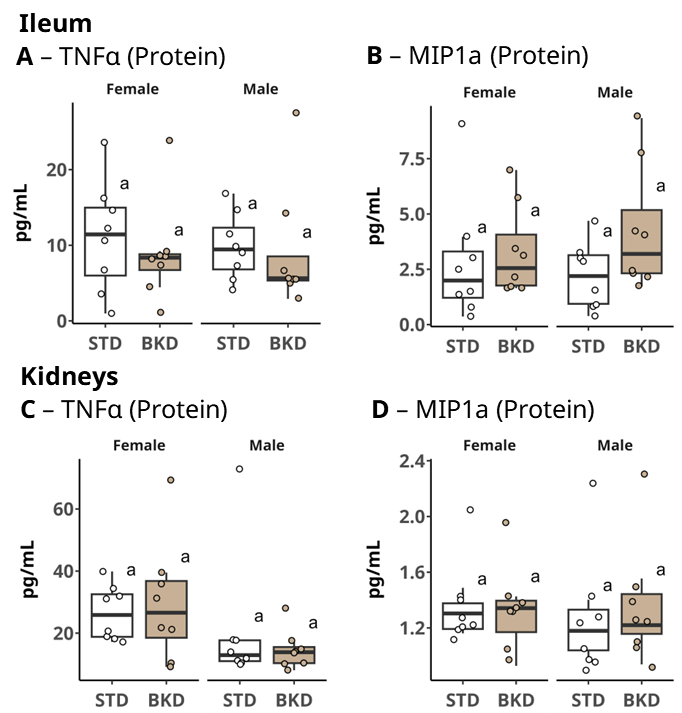


**Figure S2.** Inflammation profiling in the ileum and kidneys. **(A)** TNFα protein and **(B)** MIP1α protein expression in the ileum. **(C)** TNFα mRNA and **(D)** MIP1α protein expression in the kidneys. Bars represent mean ± SEM (n = 8 sex/group). Different letters indicate statistically significant differences among groups within each organ (Kruskal–Wallis test followed by Dunn’s post hoc test for multiple comparisons, α = 0.05).

**Table S1.** Summary of DNA Sequencing Data (raw data).

| **Sample** | **Read Count** | **Q30 (%)** |
| --- | --- | --- |
| External control | 47476 | 90.5 |
| Negative control | 995 | 80.2 |
| C0812CA | 34350414 | 92.7 |
| C0814CA | 34902416 | 92.1 |
| C0821CA | 35311442 | 92.9 |
| C0822CA | 37469534 | 92.9 |
| C0823CA | 50265014 | 92.5 |
| C0824CA | 34388616 | 93.1 |
| C0825CA | 36419156 | 92.8 |
| C0830CA | 23179636 | 90.8 |
| H2251CA | 36292688 | 92.7 |
| H2268CA | 31566698 | 92.4 |
| H2269CA | 30553442 | 92.8 |
| H2270CA | 33273068 | 92.2 |
| H2271CA | 35363118 | 92.9 |
| H2272CA | 33825938 | 92.6 |
| H2273CA | 30637580 | 92.5 |
| H2274CA | 34222386 | 92.5 |
| I0628CA | 35648224 | 92.7 |
| I0629CA | 31056904 | 92.9 |
| I0630CA | 37009380 | 92.3 |
| I0631CA | 33487972 | 92.6 |
| I0632CA | 34095264 | 92.1 |
| I0633CA | 33323468 | 93 |
| I0634CA | 35558320 | 92.5 |
| I0635CA | 28217114 | 92.1 |
| T0993CA | 27011932 | 91.4 |
| T0994CA | 38032236 | 92.9 |
| T0995CA | 31882656 | 93.2 |
| T0996CA | 31257456 | 93 |
| T0997CA | 31643972 | 92.7 |
| T0998CA | 40205554 | 92.2 |
| T0999CA | 31587048 | 92.1 |
| T1000CA | 38029322 | 92.8 |
| ZymoBiomics | 31793738 | 93.1 |

**Table S2.** Quality Control and Preprocessing Summary for 35 Sequencing Libraries (raw data).

| **Sample name** | **Raw reads (number)** | **Cleaned reads (number)** | **Cleaned reads (percent raw reads)** |
| --- | --- | --- | --- |
| External control | 23738 | 20306 | 85.54 |
| Negative control | 4975 | 3696 | 74.29 |
| C0812CA | 17175207 | 15555498 | 90.57 |
| C0814CA | 17451208 | 15109761 | 86.58 |
| C0821CA | 17655721 | 15870597 | 89.89 |
| C0822CA | 18734767 | 16884068 | 90.12 |
| C0823CA | 25132507 | 22712924 | 90.37 |
| C0824CA | 17194308 | 15723891 | 91.45 |
| C0825CA | 18209578 | 16417614 | 90.16 |
| C0830CA | 11589818 | 9246786 | 79.78 |
| H2251CA | 18146344 | 16494117 | 90.89 |
| H2268CA | 15783349 | 13873422 | 87.9 |
| H2269CA | 15276721 | 13619762 | 89.15 |
| H2270CA | 16636534 | 15086258 | 90.68 |
| H2271CA | 17681559 | 16114266 | 91.14 |
| H2272CA | 16912969 | 15360929 | 90.82 |
| H2273CA | 15318790 | 13703884 | 89.46 |
| H2274CA | 17111193 | 15238683 | 89.06 |
| I0628CA | 17824112 | 15817091 | 88.74 |
| I0629CA | 15528452 | 13900076 | 89.51 |
| I0630CA | 18504690 | 16504695 | 89.19 |
| I0631CA | 16743986 | 15022738 | 89.72 |
| I0632CA | 17047632 | 15056782 | 88.32 |
| I0633CA | 16661734 | 15141898 | 90.88 |
| I0634CA | 17779160 | 15554246 | 87.49 |
| I0635CA | 14108557 | 12423515 | 88.06 |
| T0993CA | 13505966 | 11062228 | 81.91 |
| T0994CA | 19016118 | 17121622 | 90.04 |
| T0995CA | 15941328 | 14444364 | 90.61 |
| T0996CA | 15628728 | 13984500 | 89.48 |
| T0997CA | 15821986 | 14409897 | 91.08 |
| T0998CA | 20102777 | 17879009 | 88.94 |
| T0999CA | 15793524 | 13781881 | 87.26 |
| T1000CA | 19014661 | 17159244 | 90.24 |
| ZymoBiomics | 15896869 | 14722154 | 92.61 |
